# The Efficacy and Safety of Lower-Limb Plyometric Training in Older Adults: A Systematic Review

**DOI:** 10.1007/s40279-018-1018-x

**Published:** 2018-11-02

**Authors:** Tomas Vetrovsky, Michal Steffl, Petr Stastny, James J. Tufano

**Affiliations:** 1 The Strength and Conditioning Laboratory, Department of Physiology and Biochemistry, Faculty of Physical Education and Sport, Charles University, Jose Martiho 269/31, 162 52 Prague 6, Czech Republic; 2 Department of Sport Games, Faculty of Physical Education and Sport, Charles University, Jose Martiho 269/31, 162 52 Prague 6, Czech Republic

## Abstract

**Background:**

The aging process is associated with a progressive decline of neuromuscular function, increased risk of falls and fractures, impaired functional performance, and loss of independence. Plyometric training may mitigate or even reverse such age-related deterioration; however, little research on the effects of plyometric exercises has been performed in older adults.

**Objective:**

The objective of this systematic review was to evaluate the safety and efficacy of plyometric training in older adults.

**Methods:**

Papers reporting on randomized trials of plyometric training in older adults (≥ 60 years) and published up to December 2017 were sought in the PubMed, SPORTDiscus, Scopus, and EMBASE databases, and their methodological quality was assessed using the Physiotherapy Evidence Database (PEDro) scale. A narrative synthesis of the findings is presented in this systematic review.

**Results:**

Of the 2236 identified papers, 18 were included in the review, reporting on 12 different studies with a mean PEDro score of 6.0 (range 4–7). Altogether, 289 subjects (176 females and 113 males) were included in 15 intervention groups with plyometric components (*n* = 8–36 per group); their mean age ranged from 58.4 to 79.4 years. The plyometric training lasted from 4 weeks to 12 months. Muscular strength, bone health, body composition, postural stability, and jump and physical performance were the most often reported outcomes. No study reported increased occurrence of injuries or other adverse events related to plyometric exercises.

**Conclusion:**

Plyometric training is a feasible and safe training option with potential for improving various performance, functional, and health-related outcomes in older persons.

## Key Points

**Table:** 

Plyometric training positively affects muscular strength, jump performance, and physical performance in older adults.
Given the scarcity of plyometric training research in older adults, only limited evidence demonstrates superiority of plyometric training over other types of training with similar volume and intensity.
Plyometric training was demonstrated to be a safe training option in older adults when properly programmed, especially when administered in a supervised setting.

## Background

The number of older persons—those aged 60 years or over—is expected to more than double by 2050, rising from 962 million globally in 2017 to 2.1 billion in 2050 [1]. Regardless of the statistics, the aging process often involves physiological and neuromuscular changes such as sarcopenia [2], a parallel decrease in muscular strength and power [3], progressive loss of bone mass [4], and declines in coordination and balance [5]. As the effects of these age-related illnesses become more manifest, functional performance becomes impaired [6], the risk of falls increases [7], and bones are more likely to fracture [8], ultimately making daily functional activities more difficult and possibly resulting in a loss of independence [9].

Fortunately, various types of exercise interventions can reverse or at least mitigate such age-related declines in health and daily life. For example, pilates, Tai Chi, and step training improve static and dynamic balance and reduce the risk of falls [10–12]. Furthermore, resistance training can increase physical performance and muscle strength, even in very old individuals [13–18]. Additionally, weight-bearing and impact exercises, such as jumping, have beneficial effects on bone mineral density and decrease the risk of fractures in postmenopausal women and older men [19–22].

Specifically, jumping exercises can provide a variety of stimuli, most notably impact stimuli and neuromuscular stimuli [23, 24]. In older adults, some jumps are performed with “hard landings” [25] that aim to increase osteoblast formation and ultimately increase bone health [26]. However, these impact exercises do not place a focus on neuromuscular performance, and people who participate solely in impact jumping may not be exposed to dynamic force absorption and production [27], both of which are very important in daily life. Therefore, a specific type of jump training, plyometric training, is a popular exercise technique that employs rapid eccentric motion followed immediately by a rapid concentric contraction. The quick transition from the eccentric to the concentric phase is known as the stretch–shortening cycle and is one of the underlying mechanisms of plyometric training. A typical example of a plyometric exercise is a counter-movement jump, in which a downward squatting motion is followed immediately by an explosive concentric extension of the hips, knees, and ankles. Other examples involve rope jumping, box jumping, and various types of hopping and bounding.

Although plyometric exercises were originally utilized in sports training to promote jump performance, agility, muscular power, and rapid force production [28–31], these same effects of plyometrics can be beneficial for older adults. For example, high-speed training, which is associated with increases in muscle power, has been shown to increase functional performance and health-related quality of life in older women [32, 33]. Such increases in functional performance and health-related quality of life may be partially explained by increases in rapid force production, which declines more than maximal strength [34]. In daily life, rapid force production is crucial in situations when balance needs to be corrected quickly after tripping [35, 36], and its decline may be a major contributor to the loss of independence and falling accidents and injuries in older adults [7, 37]. Additionally, both agility and lower-extremity muscle power correlate well with balance [38], and having greater agility and more powerful legs thus likely indicates improved balance, which may decrease the risk of fractures and other fall-related injuries. Therefore, if increasing or maintaining rapid force production and power output of the lower limbs can help to maintain independence and decrease the fear or risk of falling, plyometric training may help maintain or increase one’s quality of life.

Despite these potentially beneficial effects, a recent scoping review of plyometric training found that little research has been performed in older adults [39]. This might be explained by the fact that plyometric exercises often require great neuromuscular control and a substantial level of strength [40], which makes practitioners unsure about their safety and feasibility in older adults who likely do not possess the basic neuromuscular control and strength levels that have long been accepted for athletes prior to high-intensity plyometric training [40, 41]. Since this time, others have argued that only basic bodyweight movement competency should be sufficient before progressively introducing simple plyometrics within a training program [42]. In support of this, plyometric exercises have been successfully employed even in very old adults with a mean age of 79.4 years [43] and in subacute stroke patients with hemiparesis [44] without injuries or other adverse events. However, no review has explored whether plyometric training in older adults is a safe and efficacious training modality.

Recently, several systematic reviews of plyometric training interventions have been published, but these focused mostly on sport-related performance in younger athletes (vertical jump performance in female athletes [28], athletic performance in youth soccer athletes [45], motor performance in young children [46], and physical fitness in team sport athletes [47]), with one exception that determined the effect of plyometric training on bone health in children and adolescents [48]. Furthermore, a recent meta-analysis demonstrated that jumping training in adults aged ≥ 50 years is safe and has moderate effect on muscular power [49]. However, to the best of our knowledge, no review has explored the effects of plyometric training in older adults (≥ 60 years) with regard to a wide range of outcomes that can potentially improve their health and daily life. Therefore, the objective of our systematic review was to evaluate the safety and efficacy of plyometric training in older adults regarding various performance, functional, and health-related outcomes.

## Methods

The present review is reported in accordance with the PRISMA (Preferred Reporting Items for Systematic Reviews and Meta-Analyses) statement [50], and the review protocol has been registered in the international prospective register of systematic reviews (PROSPERO: CRD42018093652).

### Search Strategy

The following electronic bibliographic databases were searched: PubMed, SPORTDiscus (via EBSCO), Scopus, and EMBASE. The search terms were developed to include papers that reported on various jumping and hopping exercises even if the papers may not have explicitly mentioned ‘plyometric training’. The search terms used in PubMed are given in Table 1; the search terms were adapted as appropriate for other databases. Studies published from the inception of the databases up to December 2017 were sought, and the search was limited to papers published in English-language academic journals. Additionally, the reference lists of eligible papers and of several recently published reviews [19–22, 39] were hand-searched for further studies.

**Table 1:** Search terms for PubMed

((((eccentric AND concentric) OR (stretch AND shortening) OR (deceleration AND acceleration) OR (stretch AND elastic)) AND muscle) OR (jump* OR hop OR hops OR hopping OR skipping OR countermovement OR bounding) OR plyometr*) AND (exercis*[tiab] OR “training”[tiab]) AND (aged[mh] OR middle aged[mh] OR aging [mh] OR aging[tiab] OR ageing [tiab] OR elder*[tiab] OR older[tiab] OR geriatr*[tiab] OR postmeno*[tiab])	

From the list of potential articles, duplicates were removed and two reviewing authors (TV and MS) screened the titles and abstracts of the remaining articles to identify studies that potentially met the eligibility criteria listed in Sect. 2.2. The full texts of those potentially eligible papers were then retrieved and assessed for eligibility by the same two reviewing authors. Any disagreement was resolved through a discussion with a third reviewer (JJT).

### Eligibility Criteria

This review included randomized trials that compared plyometric training or multicomponent training with plyometric component in older adults (mean age of the randomized sample ≥ 60 years) with either a control group or another exercising group. Studies of both healthy subjects and patients with specific diseases and conditions were eligible.

Plyometric exercises were defined as eccentric loading immediately followed by a concentric contraction (commonly known as the stretch–shortening cycle) and typically involved repetitive jumping, hopping, bounding, and skipping. Therefore, single jumps with a prolonged recovery after landing were not considered to be plyometric, and studies including only such non-plyometric exercises were not included in this review.

Cross-sectional studies, review papers, and studies with only a single exercise session were excluded. Studies with training programs with only a negligible plyometric component (< 10% of the training volume, either stated by the original authors or estimated by the reviewing authors of the current paper [TV, MS, and JJT]) and studies with ambiguous methodology sections from which the plyometric nature of the exercises could not be determined were also excluded.

### Quality Assessment

The Physiotherapy Evidence Database (PEDro) scale was used to assess the methodological quality of the included studies [51]. The assessment was performed by one of the authors of this review (TV) and the resulting scores were compared with the available scores in the PEDro database where possible. Any incongruities were then discussed and resolved with two other authors (MS and JJT).

### Data Extraction and Synthesis

Data were extracted by two of the reviewers (MS and TV), using an Excel^®^ spreadsheet (Microsoft Corp., Redmond, WA, USA). Extracted information included study design, study population (number of participants, age, sex, healthy vs. clinical population), details of the training program (length, frequency, volume, intensity, plyometric alone vs. multicomponent training, supervised vs. home sessions, type of exercises), details of comparison group, outcomes and length of follow-up, and safety measures.

Given the relative paucity of plyometric training in older adults unveiled during our pilot literature searches, specific outcomes were neither required nor ignored for the sake of this review. Rather, all performance, functional, and health-related outcomes were extracted and synthesized. Both narrative and quantitative syntheses of findings from the included studies, structured around the target population characteristics, type of outcome, and intervention content, are provided in this review.

## Results

### Study Selection

**Fig. 1:**
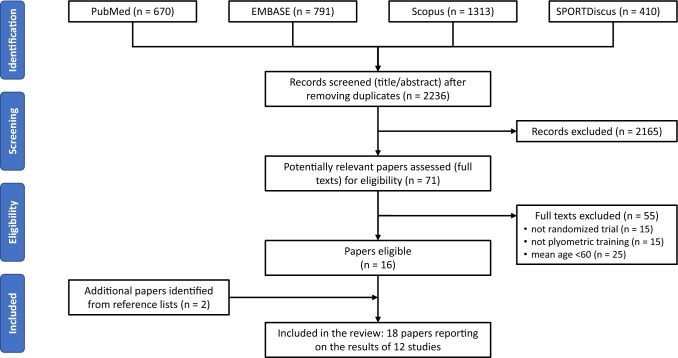
Flowchart illustrating the different phases of the search and study selection

The database search yielded 2236 different papers (Fig. 1), 16 of which were eligible. Screening of their reference lists identified two more eligible papers. Hand searching of other recent plyometric reviews [28, 39, 47] did not reveal any additional papers. Altogether, the 18 included papers [43, 52–68] report on the results of 12 different studies, as depicted in Table 2.

**Table 2:** Summary of the reviewed studies

Study^a^	Groups (sample size)	Mean age (SD) of the whole sample	Participant characteristics	Length of the intervention (length of follow-up)	Plyometric intervention description	Weekly volume
Allison et al. [52, 53]	Unilateral intervention of one-leg hopping (35)	69.9 (4)	Healthy males	52 weeks	Unilateral hopping exercises (on a hard, even surface while barefoot) with multidirectional hops introduced in the ninth week	Frequency progressing from 3 to 7 times per week, number of sets progressing from 3 to 5 sets of 10 hops interspersed with 15 s rest period
Bolton et al. [54]	Combined exercises (19) and control (18)	60.3 (5.6)^b^	Postmenopausal females with osteopenia	52 weeks	Supervised resistance training, impact loading and balance exercises with individualized progression of the duration and intensity plus home program consisting of daily jumps introduced 12 weeks after commencing the intervention	3 times per week for 60 min (supervised) plus up to 10 jumps, 3 times daily at home
Karinkanta et al. [55–58]	Resistance (37), balance-jumping (35), combination of resistance and balance-jumping (36), and control (36)	72.9 (2.3)^b^	Healthy females	52 weeks (with follow-ups 12 months and 5 years after cessation of the intervention)	Balance, agility, and impact exercises including jumps in the form of regularly alternating aerobics or step aerobics training programs with gradually increasing difficulty of movements	3 times per week, 40–50 min
Bolam et al. [59]	High-dose jumping (13), low-dose jumping (15), or control (14)	62.1 (6.9)^b^	Healthy males	40 weeks	Two supervised sessions of combined impact-loading (multidirectional jumping, drop jumping, and bounding over soft foam hurdles) and upper-body resistance exercise. Two home sessions of multidirectional and vertical jumps	4 times per week, 80 (high-dose) or 40 (low-dose) jumps per session in sets of 5–14 repetitions with rest periods of 1 min between sets
Marín-Cascales et al. [60, 61]	Whole-body vibration (15), multicomponent training (13), and control (10)	60.0 (6.3)	Postmenopausal females	24 weeks	Multicomponent training consisted of aerobic activity and a series of drop jumps with progressive load imposed by increasing height from 5 up to 25 cm	3 times per week, 30–60 min, 4–6 sets of 10 jumps
Ramírez Villada et al. [62]	Jump training (15), concurrent jump and machine training (15), and control (15)	59.5 (6.4)	Healthy females	22 weeks	Various multiple jumps ranging from basic jumps with the support of both lower limbs to jumps with turns and obstacles	2 times per week with the total repetitions per week ranging from 128 to 144
Correa et al. [63]	Strength (14), power (13), plyometric (14) exercises, and control (17)	67.0 (5.0)	Healthy females	12 weeks	Leg press, knee extension, and knee flexion in the first 6 weeks. In the second 6-week phase, leg press was replaced with lateral box jump exercise on a box with a height of 10–30 cm	3–4 times per week, 3–4 sets (15–20 sec)
Piirainen et al. [64]	Plyometric (9) and pneumatic (11) training	63 (2)^b^	Healthy males	12 weeks	Continuous countermovement jumps using a sledge apparatus that enables drop jumps to be performed more safely than standard vertical jumping	2–3 times per week, 5 sets of 6 repetitions
Rantalainen et al. [65, 66]	Hopping (10) and control (10)	73 (4)^b^	Healthy males	11 weeks	Continuous bilateral hopping on the balls of the feet at submaximal intensity (75–90% of the maximal ground reaction force)	3 times per week, 5–7 sets (10 sec)
Váczi et al. [67]	Stretch-shortening cycle contractions (8) and isokinetic eccentric contractions (8)	64.4 (4.1)^b^	Healthy males	10 weeks	Unilateral knee extensions with both legs in which a dynamometer rapidly applied a preset amount of energy to stretch the quadriceps and subjects were instructed to resist the rotating lever arm maximally, stop it within the shortest range of motion, and then extend the knee without a time delay and as fast as possible	2–3 times per week, 4 sets of 8–14 repetitions with rest periods of 2 min between sets
Cakar et al. [43]	Combined exercises (30) and combined exercises plus jumping (36)	79.4 (5.4)^b^	Males (*n* = 26) and females (*n* = 40) living in a long-term care facility	6 weeks	10 min of vertical jumping with self-paced intensity. The participants were encouraged to maintain a high level of effort. Combined exercises included stretching, strength, and aerobic exercises	3 times per week, 30–45 min of combined exercises plus 10 min of jumping
Park et al. [68]	Jumping plus therapeutic exercises (16) and therapeutic exercises alone (15)	76.7 (8.6)^b^	Males (*n* = 14) and females (*n* = 17) living in a long-term care facility	4 weeks	At least 30 jumps of > 2 cm above the ground per session. The jumping frequency was not limited. Therapeutic exercises included stretching, fast walking, squats, etc.	5 times per week, 40 min of therapeutic exercises plus 20 min of jumping exercises

*SD* standard deviation

^a^Studies are ordered by (1) training duration; and (2) author name

^b^Mean age of the whole sample was not reported; therefore, mean age of the plyometric training group is presented

### Methodological Quality

The mean PEDro score of the included studies was 6.0, with nine of 12 studies receiving high-quality ratings of ≥ 6 (Table 3). Due to the nature of the interventions, none of the studies blinded subjects and therapists, but two studies scored positive for blinding of assessors [52, 54], and six studies satisfied the item for concealed allocation [43, 52, 54, 55, 65, 68].

**Table 3:** Physiotherapy Evidence Database (PEDro) scores of the included studies

Study^a^	Eligibility criteria	Randomization	Concealed allocation	Similar group baselines	Blinding of all subjects	Blinding of all therapists	Blinding of all assessors	Dropout < 15%	Intention-to-treat method	Statistical between-group comparison	Point measures and measures of variability	Score
Allison et al. [52]	+	+	+	+	−	−	+	−	+	+	+	7
Bolton et al. [54]	+	+	+	−	−	−	+	+	+	+	+	7
Karinkanta et al. [55]	+	+	+	+	−	−	−	+	+	+	+	7
Park et al. [68]	+	+	+	+	−	−	−	+	+	+	+	7
Bolam et al. [59]	+	+	−	+	−	−	−	+	+	+	+	6
Cakar et al. [43]	+	+	+	+	−	−	−	−	+	+	+	6
Correa et al. [63]	−	+	−	+	−	−	−	+	+	+	+	6
Piirainen et al. [64]	−	+	−	+	−	−	−	+	+	+	+	6
Rantalainen et al. [65]	+	+	+	+	−	−	−	−	+	+	+	6
Marín-Cascales et al. [60]	+	+	−	+	−	−	−	−	+	+	+	5
Váczi et al. [67]	+	+	−	−	−	−	−	+	+	+	+	5
Ramírez Villada et al. [62]	−	−	−	+	−	−	−	−	+	+	+	4

^a^Table consists only of the original research experiments, not including subsequent publications reporting secondary or follow-up data. Studies are ordered by (1) final score; and (2) author name

Of the 12 studies in our review, nine had already been assigned scores in the PEDro database. Within those nine studies, there were 14 incongruities with our scores. Of those, in two cases we changed the scores in line with the PEDro database, and in the remaining 12 cases we retained our original scores. Of note were seven incongruities in scoring the ‘intention to treat’ item that PEDro raters scored negative, whereas we chose to award them positive scores.

### Study Characteristics

The included papers were published between 2007 and 2017 in 15 journals. Of the 12 studies, six were conducted in Europe (UK, Spain, Hungary, and three in Finland), and two each in Australia, South America (Colombia, Brazil), and Asia (Turkey, Korea).

Most of the studies compared two groups, either an exercise intervention and non-exercising control (*n* = 2), or exercise with and without additional jumping (*n* = 2), or two different exercise interventions (*n* = 2). Three studies compared two experimental groups with a control group, and two studies compared three experimental groups with a control group. In addition, one study was designed as a randomized unilateral intervention comparing an exercise leg with a control leg [52].

Altogether, there were 15 different intervention groups that included a plyometric component, including 289 subjects in total (*n* = 8–36 per group); of these, 176 were females and 113 were males. The mean age in the intervention groups ranged from 58.4 to 79.4 years. Most studies recruited exclusively either males (*n* = 5) or females (*n* = 5), with only two studies recruiting both males and females [43, 68]. The majority of studies recruited healthy, community-dwelling older adults (*n* = 9), one study recruited postmenopausal women with osteopenia [54], and two studies recruited older residents of long-term care facilities [43, 68].

### Plyometric Training

Of the included studies, the interventions with a plyometric component lasted from 4 weeks [68] to 12 months [52, 55]. The number of training sessions per week ranged from two to seven, and often varied throughout the course of the intervention period. In all studies, the training sessions were supervised, but in two studies subjects also completed training sessions at home [54, 59].

Some studies were designed so that the effect of plyometric training could be evaluated in isolation, i.e., they either had a group allocated to purely plyometric exercises [52, 64, 65, 67] or they compared groups performing exactly the same non-plyometric exercises with one of these groups also performing additional exercises that were plyometric [43, 68]. In other studies, the plyometric exercises were part of a multifaceted exercise intervention that included resistance training [54, 59, 63], aerobic training [60], balance exercises [54, 55], or agility exercises [55].

In most of the studies, the plyometric exercises consisted of various types of jumping, bounding, and hopping, both unilateral and bilateral. The two exceptions were studies by Váczi et al. [67], who had their subjects perform unilateral knee extensions on an isokinetic dynamometer that consisted of a rapid stretch–shortening cycle action, and by Piirainen et al. [64], who used a sledge apparatus that supposedly enabled drop jumps to be performed more safely than standard vertical jumping.

### Study Outcomes

Various outcomes evaluated in the studies are summarized in Table 4 together with the assessment methods. Muscle strength, assessed in eight studies, was the most often reported outcome, followed by bone health and body composition, evaluated in six studies. Effects on postural stability, jump performance, and physical performance were reported in five studies.

**Table 4:** Main plyometric-related outcomes evaluated in the studies

Outcome	Measure	Overall effect	References^a^
Muscular strength	Hip flexion, knee extension, and ankle plantar- and dorsiflexion assessed by a handheld dynamometer	Positive	[68]
	Rate of torque development of the quadriceps femoris assessed by a dynamometer	Positive	[67]
	Knee extension test (1RM) assessed on a knee extension machine	Positive: similar to others without plyometrics	[63]
	Leg extensor force assessed by a leg press dynamometer	Positive: similar to others without plyometrics, but disappeared after 1 year	[55, 56]
	Plantar flexion and knee extension maximal voluntary contractions assessed by an isokinetic dynamometer	Positive: similar to others without plyometrics	[64]
	Maximal voluntary isometric concentric and isokinetic eccentric torque, work during a concentric contraction, work during a plyometric contraction, time of the plyometric contraction	Positive: similar to others without plyometrics	[67]
	Isokinetic knee extension, dorsal and plantar flexion, and eversion and inversion assessed by an isokinetic dynamometer	Likely positive: similar to others without plyometrics	[60, 61]
	Maximum voluntary isometric dominant knee extension and back extension assessed by an isokinetic dynamometer; dominant leg hip abduction and hip flexion assessed by a handheld dynamometer	Unclear: likely positive effect, but similar to control group	[54]
	Leg press (1RM)	No effect	[59]
Bone health	BMD and content of proximal femur assessed by DXA	Positive	[52]
	BMD of the total hip and lumbar spine assessed by DXA	Positive	[54]
	Cortical, trabecular, and integral bone mineral content at traditional regions of interest within the proximal femur assessed by QCT	Likely positive: site-specific, but not global, increases compared to control group	[53]
	BMD of the hip and trochanter assessed by DXA	Unclear: possible positive effect for high-dose group	[59]
	Total hip, femoral neck, trochanter, and lumbar spine BMD assessed by DXA	No effect	[59]
	Bone turnover markers (bone-specific alkaline phosphatase and C-terminal telopeptide of type 1 collagen)	No effect	[59]
	BMD and content of the right proximal femur assessed by DXA	No effect	[55, 56]
	QCT performed at the distal sites (trabecular bone) and at midshaft (cortical bone) of the right radius and tibia	No effect	[55, 56]
	BMD assessed by DXA	No effect	[60, 61]
	Bone turnover markers (C-terminal propeptide of type 1 collagen, bone-specific alkaline phosphatase, and C-terminal telopeptide of type 1 collagen)	No effect	[65]
Body composition	Body mass, lean body mass (calculated by the Rose formula), and fat mass (estimated by Brozek’s equation)	Positive	[62]
	Anatomical cross-sectional area measured by magnetic resonance imaging on the components of the quadriceps	Positive: same as others without plyometrics	[67]
	Total fat mass, lean mass, and body fat percentage assessed by DXA	Likely positive: similar to others without plyometrics, but better than control	[60, 61]
	Muscle thickness (vastus lateralis, medialis, and rectus femoris) assessed by ultrasonography	Unclear: positive effect for vastus lateralis, which was similar to others without plyometrics; no effects for other muscles	[63]
	Fat and lean tissue mass of the whole body assessed by DXA	No effect	[52]
	Whole-body lean mass, fat mass, percentage body fat assessed by DXA	No effect	[59]
	Skinfolds (biceps, triceps, subscapular, suprailiac) measured by Holtain calliper	No effect	[62]
Postural stability	Berg balance test; dynamic balance on a balance platform	Positive	[43]
	Berg balance test; postural sway on a force plate	Positive	[68]
	Dynamic balance on a balance platform	Unclear: directional-specific and time-specific effects present, some similar to others without plyometrics	[64]
	Static and dynamic balance in barefoot standing with eyes open on a balance platform	No effect	[54]
	Static posturography on a force plate assessed by center-of-pressure velocity	No effect	[65]
Jump performance	Countermovement jump on a force plate	Positive	[63]
	Maximal drop jump performance and optimal dropping height on a sledge ergometer	Positive: time-specific changes, but similar to others without plyometrics	[64]
	Ground contact time, ground reaction force, and ankle joint stiffness during repeated two-legged hopping on a force plate, reactive strength index, jump height	Positive: time-specific changes, but generally better for plyometric group	[66]
	Peak vertical ground reaction forces during take-off and landing averaged over 10 hops on a force plate	Likely positive	[52]
	Squat jump, countermovement jump, and countermovement jumps with arm swing on an OptoGait system (jump height, take-off velocity, flight time)	Likely positive: data in original article’s tables somewhat confusing	[62]
	Jump height and ground reaction forces determined from 2–4 maximal jump efforts during repetitive two-legged hopping on a force plate	No effect	[65]
Physical performance	30-s sit-to-stand test	Positive	[63]
	A standardized figure-of-8 running test around two poles placed 10 m apart	Positive: similar to others without plyometrics, but effect may disappear if training is not continued	[55, 56]
	Timed up-and-go test	Positive	[68]
	Velocity-agility test (30 m) and shuttle run test (30 m)	Likely positive: effect similar to others without plyometrics	[62]
	6-m fast walk	Unclear: positive effect for moderate-dose group, but no effect for high-dose group	[59]
	6-m usual walk, backwards tandem walk, stair climb, or sit-to-stand test	No effect	[59]
Quality of life /daily function	Health-related quality of life, assessed by the SF-36 survey	Positive: to the same degree as others without plyometrics	[43]
	Finnish Physical Functioning Scale of the SF-36, fear of falling	Positive: effect likely to disappear if training stops	[55–57]
	Quality of life assessed by the Assessment of Quality of Life survey, mental health assessed by the SF-36 survey, and physical activity assessed on the Physical Activity Scale for the Elderly questionnaire	Likely positive: all variables generally better maintained in exercise group	[54]
	Physical activity measured by the Godin Leisure Time Exercise Questionnaire	No effect	[59]
Other	Muscle onset latency and reaction time assessed by electromyography	Positive	[63]
	Capillary glucose	Positive	[62]
	Fractures and injurious falls experienced during a 5-year follow-up period	Positive	[58]
	Maximal muscle activity by maximal electromyography root mean square (vastus lateralis, medialis, and rectus femoris).	Likely positive: effect similar to others without plyometrics	[63]
	Kinematics of ankle and knee joint angles	Unclear: possibly a minor effect, data varied at different training times and hopping intensities	[66]
	Gastrocnemius medialis–muscle fascicle and its outer Achilles tendon length changes examined by ultrasonography	Unclear: possibly a minor effect, data varied at different training times and hopping intensities	[66]
	H-reflex during standing rest and drop jumps, EMG activity during drop jumps	Unclear: plyometric group likely experienced positive explosive/reflex-specific changes, but not during slow/maximal strength tasks	[64]
	EMG activities of calf muscles	No effect	[66]
	Testosterone, estradiol, vitamin D levels, blood calcium levels	No effect	[59]
	Total testosterone and cortisol serum levels	No effect	[67]

^a^References are ordered by (1) effect; and (2) author name

*1RM* 1-repetition maximum, *BMD* bone mineral density, *DXA* dual-energy X-ray absorptiometry, *EMG* electromyography, *QCT* quantitative computed tomography, *SF-36* Rand 36-Item Health Survey

The outcomes were usually assessed after the end of the intervention. In some papers, authors also reported interim results. For example, Piirainen et al. [64] assessed outcomes of a 12-week intervention at 4, 8, and 12 weeks. Similarly, Marín-Cascales et al. [61] reported the 12-week results of a 24-week intervention in a separate paper. On the other hand, Karinkanta et al. [56] reported on the maintenance of exercise-induced benefits 1 year after the cessation of a 12-month intervention. In addition, the same investigators conducted a 5-year register-based follow-up study to assess the risk of injurious falls and fractures long after the intervention ended [58].

Based on the quantitative data presented in each study, a qualitative summary of the data is presented in Table 4. Although the outcomes of some studies were quite straightforward and easy to describe (e.g., Park et al. [68] found that when a plyometric group and a control group performed the same therapeutic exercises, but the plyometric exercising group also included jumping, the plyometric group increased hip, knee, and ankle flexion and extension strength to a greater extent over a 4-week period), the results of other studies were much more difficult to describe. For example, the study by Correa et al. [63] had two study phases. The first phase compared a non-exercising control group (*n* = 17) to a resistance-exercise group (*n* = 41), and the second phase compared a control group (*n* = the same 17) to three different subdivided resistance-training groups: traditional strength training (*n* = 14), power training (*n* = 13), and rapid strength training that included plyometric exercise (*n* = 14). During the second phase of this study, the rapid strength group increased the thickness of the vastus lateralis muscle, but no change was present in the vastus medialis or rectus femoris: similar changes were seen in the other resistance-training groups. Since muscle thickness increased in the plyometric group, but only in one of the three tested muscle groups, and to a similar degree as other training groups, it is *unclear* whether the addition of plyometrics played a significant role in increasing muscle thickness. In the same study, the mean square root of electromyographic signals increased in the vastus lateralis and medialis, but not in the rectus femoris; a pattern that was the same in all three groups. In this case, since the changes were present in two of the three tested muscle groups, there was *likely* a positive effect of plyometric exercise, but we still cannot be 100% sure as the increase was similar to the other training groups. Therefore, to maintain the simplicity and readability of the results in Table 4, the overall results for a given variable (e.g., mean square root for multiple muscle sites, possibly across multiple timepoints) were combined and then presented as either a ‘positive’, ‘likely positive’, ‘unclear’, or ‘no effect’ result. A ‘positive’ result indicates that nearly all of the data displayed a positive result at nearly all of the timepoints assessed within the study period. A ‘likely positive’ result indicates that over half of the data displayed a positive result at the majority of the timepoints assessed within the study period. An ‘unclear’ result indicates that less than half of the data displayed a positive result within the study period, but no negative results were present. A ‘no effect’ result indicates that the variable did not change, for better or worse, within the study period. As such, no unwarranted, or ‘negative’, effects were present for any of the tested variables. Nevertheless, although the simplified qualitative results are displayed in Table 4, quantitative values are expressed where appropriate throughout Sect. 4.

### Safety

Five studies reported that there were no injuries or other adverse events related to the exercise protocol or testing [43, 52, 59, 60] and four studies did not report on possible adverse events at all [63, 64, 67, 68], likely indicating that no adverse events occurred.

Karinkanta et al. [55] reported that four exercisers fell during supervised exercise sessions, but returned to the training classes within 2 weeks. They also noted that during the intervention, 14 participants from the training groups consulted the attending physician (one knee ligament injury, one quadriceps femoris injury, and ten reports of overuse symptoms), and one participant was taken to the hospital due to acute low back pain. Three of these participants did not return to the training classes, but it is not clear whether they were from the group that included plyometric training. Despite these occurrences, there were no differences in the number of monthly reported health problems between exercisers and controls [55]. Moreover, the investigators found that women from the multi-component training group combining resistance and balance-jumping exercises had a reduced incidence of injurious falls during the 5-year post-intervention period in comparison with the control group [58].

In the study by Bolton et al. [54], the authors observed similar rates of adverse events, including falls in both exercise and control groups, and concluded that the exercise intervention was safe despite three participants attributing ankle or knee pain to the exercise. In addition to supervised sessions, this study also included home-based unsupervised sessions, but the authors did not specify whether the adverse events occurred during supervised or unsupervised sessions. Rantalainen et al. [65] reported that one subject dropped out of the intervention group (*n* = 13) due to a musculoskeletal injury that was likely related to the intervention.

## Discussion

To the best of our knowledge, this is the first systematic review to evaluate the efficacy and safety of plyometric training in older adults. The results indicate that plyometric exercises might have potential for improving various performance (muscular strength, jump and physical performance), functional (postural stability, daily function), and health-related (bone health, body composition) outcomes in older persons (Table 4).

Despite a recent proliferation of published articles on the effect of plyometric training in various populations [39], we identified only 12 randomized trials (289 subjects) that examined the effect of plyometric exercises in older adults. In addition, most of the trials were relatively small, with the largest one including only 36 subjects. Furthermore, in only six studies [43, 52, 64, 65, 67, 68] could the effect of plyometric exercises be evaluated in isolation and, of those, only two studies [64, 67] compared plyometric exercises with an alternative form of exercise with equalized volume. Therefore, it would be scientifically unjustifiable to draw conclusions related to the effects of plyometric training alone versus plyometric training combined with other exercise methods. Given the potential benefits of plyometric training in older adults and the small number of studies allowing for direct comparisons between exercise modes, more research with larger sample sizes and well-designed active control groups is needed in this population.

With one exception, the studies identified in this review recruited healthy older adults. Thus, in future studies, it would be worthwhile to verify these findings in chronically ill older patients, who may respond differently to plyometric exercise and may require unique safety precautions.

In contrast to the findings of the recent scoping review that reported that less than one-quarter of plyometric jump training studies included females [39], we found that females constituted the majority of subjects (176 females and 113 males), which might be explained by increased interest by researchers in the positive effects of jumping exercises on bone composition in postmenopausal women. Although many other studies have investigated the effects of weight-bearing and impact exercises on bone mineral density, especially in women, those studies were excluded from our review because they did not include a plyometric component.

The methodological quality of the studies in this review was good (mean PEDro score of 6.0), at least in comparison with other reviews of plyometric training in which the PEDro score ranged from 4.5 to 5.25 [28, 45, 46]. To further improve the methodological quality of future studies, researchers should consider the blinding of assessors and aim for effective allocation concealment. Some of the papers notably omitted important training descriptors. For example, only a handful of the papers described exercise intensity [59, 60] or mentioned the type of training surface [53]. Reporting on these intervention details is crucial for leveraging the findings of such studies for future research and practice.

### Interpretation of Study Results

#### Muscular Strength

The strength of various leg muscle groups was reported as an outcome in eight studies [54, 55, 59, 60, 63, 64, 67, 68], primarily using dynamometry. While the majority of these studies [55, 60, 63, 64, 67, 68] found an improvement in muscle strength when comparing a plyometric or combined training with a control group, comparisons between different training modalities yielded ambiguous results. For example, a study that compared resistance training, balance-jumping training, a combination of resistance and balance-jumping training, and a control group found that relative isometric leg press force improved in the resistance and combination (+ 21.7% [+ 3.6 N/kg], effect size (ES) 0.86, *p* < 0.01) groups, but not in the balance-jumping group [55]. Similarly, in a study comparing plyometric and pneumatic power training two to three times per week, the pneumatic training group showed significantly greater rapid knee extension torque production after only 4 weeks of training (*p* < 0.01), while the plyometric group showed a significant change only after 12 weeks (*p* < 0.01) [64]. While a frequency of two to three sessions per week did not increase strength after 4 weeks in that study, another study showed that 4 weeks of jump training five times per week was sufficient to increase hip extension strength (+ 49% [+ 8.5 kg], ES 1.67, *p* < 0.001) [68]. Together, these results suggest that training duration, frequency, and volume are important variables that need to be considered when designing plyometric interventions for older adults [39, 69].

Yet another study compared the effect of isokinetic eccentric actions and stretch–shortening cycle (plyometric) contractions on quadriceps strength [67]. Both training programs produced similar improvements in maximal voluntary isometric and eccentric torque and stretch–shortening cycle function. However, the rate of torque development during isometric contraction increased only after plyometric exercise (+ 29% [+ 0.42 Nm ms^−1^], ES 0.55) [67]. Therefore, according to the studies included in this review, it is likely that plyometric training directly or indirectly increases muscular strength in older adults, but probably not to the same magnitude as resistance training [70, 71]. Additionally, limited evidence [67] suggests that plyometric exercises are superior to eccentric training in improving explosive muscle strength, which is a key deficiency of aging muscle. Of the many possible mechanisms underpinning strength adaptations that occur after plyometric training, the inhibition of Golgi tendon organs combined with repeated activation of muscle spindles may be the most likely explanation [42]. As muscle spindles are stretched during plyometric training, a neuromuscular reflex likely occurs, which may activate higher threshold motor units that would normally not be used [72, 73]. Long-term exposure to such stimuli may decrease neuromuscular inhibition, which would likely result in greater muscle activity and, in turn, greater strength. However, the myriad of mechanical and physiological variables that contribute to strength adaptation are extremely complex and would require greater elaboration, which is outside the scope of this review. Nevertheless, the data extracted from the studies of this review indicate that plyometric training likely increases muscle strength, with no studies indicating that muscle strength decreased as a result of plyometric training.

#### Bone Health

Of the six studies [52, 54, 55, 59, 60, 65] assessing bone health, usually by dual energy X-ray absorptiometry (DXA), only two showed positive results [52, 54]. A 12-month study of high-impact unilateral exercise (up to 50 multidirectional hops a day, 7 days a week) resulted in significant improvements in femoral neck bone mineral density (+ 0.6% [+ 0.006 g/cm^2^], ES 0.34, *p* < 0.05), bone mineral content (+ 0.7% [+ 0.04 g], ES 0.30, *p* < 0.05), and geometry [52]. Yet another study comparing a 52-week multicomponent intervention with non-exercising controls found a non-significant benefit of exercise on mean total hip bone mineral density (+ 0.4% [+ 0.003 g/cm^2^], ES 0.04); however, this was significantly greater than in the control group (*p* < 0.05) [54]. Other studies did not find a significant effect of exercise on bone composition [55, 59, 60] or bone turnover [59, 65]. Of note, in both studies showing positive results [52, 54], the length of the intervention was 52 weeks, while in the remaining studies, the length of the intervention was shorter (11–40 weeks), with the exception of Karinkanta et al. [55], which also lasted 52 weeks but only included a minor plyometric component. Thus, these results are in line with previous findings that sufficient training duration (and volume) are required to achieve significant improvement in bone health [74].

As these data indicate limited benefits of plyometric training for improving bone health, at least in the short-term, it is important to note that there is likely a trade-off between training to enhance neuromuscular performance and training for bone health. For example, studies included in the present review must have included plyometric training characterized by a rapid eccentric muscle action followed by a forceful and rapid concentric action. Consequently, impact forces are largely absorbed during the eccentric phase of landing, and the resultant elastic energy is then coupled with concentric force to execute the following jump, ultimately resulting in very little impact compared to jumps with “hard landings” [75]. To achieve harder landings, subjects are actually instructed to jump and land as heavily as comfortably possible, likely with the legs in a straighter position, without purposefully and eccentrically absorbing force [76]. As a result, it is likely that these impact forces are much greater than those experienced during plyometric training, where the initial impact forces are better absorbed through flexion of the hips and knees. Therefore, although plyometric training likely does not play a large role in increasing bone health, it should not be confused with jump training that includes hard landings and higher impact forces, which are likely to be more effective at increasing bone health [76]. Also, it is important to keep in mind that bone health naturally decreases in older adults, and although the findings of this review indicate that plyometric exercise may not *increase* bone health per se, any maintenance of bone health should still be considered a positive clinical outcome. Therefore, although there are data presented here and in Table 4 indicating that there may not have been a ‘positive effect’, as seen for other variables, the fact that there were no ‘negative effects’ of plyometric training on bone health is clinically and practically significant.

#### Body Composition

The body composition category includes assessments of either whole-body masses (four studies [52, 59, 60, 62]) or thickness of quadriceps muscles (two studies [63, 67]). Of the three studies assessing total lean and fat mass by DXA, two studies failed to demonstrate any improvement [52, 59] and one study showed that in the group with a plyometric component, fat mass decreased (– 5.4% [– 1.7 ± 2.0 kg], ES 0.28, *p* < 0.01) more than in a non-exercising control group (*p* < 0.001) but similarly to that of another group that completed non-plyometric exercises [60]. Interestingly, the study by Ramírez Villada et al. [62] showed that the percentage of muscle mass increased when calculated by body composition equations (+ 5.2% [+ 1.9], ES 0.48, *p* < 0.05) but did not observe any effect on absolute skinfold measurements measured by Holtain callipers. Regarding quadriceps thickness, one study showed an increase (+ 2.1% [+ 106 mm^2^], ES 0.12), which was the same as in the comparative exercise group [67], and the results of another study were unclear [63]. Additionally, it is important to note that the multi-factorial nature of the exercise programs utilized in many of these studies likely meant that the researchers were interested in the effect of the exercise programs as a whole on body composition. As such, if changes in body composition are desired, plyometric training is likely not to be the primary exercise choice for inducing changes in body composition but may be included in a periodized program to result in additional functional adaptations that may not arise from other forms of exercise interventions. Therefore, the results indicate that, similar to any other physical activity, plyometric training is associated with changes in body composition, but its effects are not likely different from those of other exercises of similar volume and intensity.

#### Postural Stability

Postural stability was assessed in five studies [43, 54, 64, 65, 68], either by various balance platforms (both static and dynamic) or by a functional test (Berg balance test). Two studies that evaluated additional jumping exercises in addition to a combined training program demonstrated improvements of various stability scores, such as overall stability where a negative value is a positive finding (– 34.0% [– 1.02 ± 0.29], ES 0.83, *p* < 0.0001) [43] and Berg Balance score (+ 16.6% [6.6 ± 2.7], ES 1.69, *p* < 0.001) [68]; these improvements were greater in the group with additional jumping than in the group with the combined training program alone (*p* < 0.05 and *p* < 0.001, respectively). However, two other studies that compared an exercise program with non-exercising controls failed to show any between-group differences [54, 65]. This is surprising and contrary to the findings of others [77]. Bolton et al. [54] explained the lack of effect as an insufficient intensity of the intervention and partially unsupervised home-based training with low adherence to exercise. Similarly, insufficient volume, insufficient intensity, or a combination of both can likely explain the lack of effect in the study by Rantalainen et al. [65], as their intervention was not effective at improving any of the study outcomes. Therefore, it appears as though plyometric training positively affects postural stability provided the training program has sufficient volume and intensity. In practice, increasing static stability, dynamic postural stability, or both may translate into better balance during activities of daily living. As a result, an older adult’s fall risk and fear of falling may decrease, which in turn may lead to increased levels of habitual physical activity [78, 79] and reduced disability and morbidity [80].

#### Jumping and Power-Based Measures

Jump performance was usually assessed on a force plate and was reported in five studies [52, 62–64, 66]. In an 11-week study, hopping training improved jump height (*p* < 0.01) in older men by decreasing the contact time (*p* < 0.05) and increasing the ground reaction force (*p* < 0.01) and reactive strength index (*p* < 0.01) [66]. Furthermore, a study comparing three exercise modalities found improved counter-movement jump height (+ 25%, *p* < 0.05) in older women whose training included plyometric jumps in comparison with women in traditional resistance training and power training groups (*p* < 0.05) [63]. Lastly, another study reported significant improvements in explosive strength measured by the subjects’ response to various types of jumps (e.g., height of countermovement jump with arm swing improved by 30% [+ 4.5 cm], ES 1.17, *p* < 0.05) in training groups that included jump training [62]. Therefore, according to the principle of specificity, it is not surprising that jumping exercises have a large impact on jump performance, especially compared with other types of training that are not task specific. Although jumping is unlikely to be part of daily life for older populations, its strong relationship to other performance measures highlights its use in scientific research. Specifically, jump and power-based performance are positively related to physical function [81] and quality of life [82], and inversely related to chronic diseases such as osteoarthritis, diabetes mellitus, and cardiovascular diseases [83]. Therefore, implementing plyometric training to increase lower-limb power output likely results in positive adaptations that reach far beyond the realm of jumping and other force plate measures.

#### Physical Performance

To evaluate physical performance, the studies used various tests (30-s sit-to-stand test, figure-of-8 running test, timed up-and-go test, 6-m walk, stair climb), with mostly positive effects. For example, in the Park et al. [68] study, the subjects improved in the timed up-and-go test (– 32% [7.3 ± 6.5 s], ES 0.87, *p* < 0.001) after only 4 weeks of an intervention that combined therapeutic exercises with jumping, and this improvement was significantly greater than in the group with therapeutic exercises alone (*p* < 0.01). Similarly, Correa et al. [63] showed that older females participating in a 12-week plyometric training program improved in the 30-s sit-to-stand test (+ 17%, *p* < 0.05), an improvement that was significantly greater than in the group performing traditional strength training (*p* < 0.05). Yet another study reported a significant improvement in a standardized figure-of-8 running test in groups that performed balance-jumping exercises alone (– 5.8% [– 1.2 s], ES 0.41, *p* < 0.01) or in combination with resistance training (–8.1% [– 1.7 s], ES 0.62, *p* < 0.001), but not in a resistance training-only group [55]. The improvement was maintained at a follow-up 12 months after the end of the intervention [56]. However, in this study, the plyometric (i.e., jumping) component formed only a minor part of the balance-jumping training, and thus it is not clear whether the improvement can be attributed to the plyometric component or rather to the balance-specific exercises. Therefore, it seems that plyometric training not only improves physical performance, but in specific tests, it may even be superior to other types of training.

#### Other Measures and Considerations

Four studies [43, 54, 55, 59] used various questionnaires to assess health-related quality of life and/or daily function. Their results were more or less positive, but did not show any superiority of plyometrics over other types of training. Three studies [63, 64, 66] employed electromyography; of note is the study by Correa et al. [63], who demonstrated that plyometric training improves muscle onset latency (– 30% [– 88 ms], ES 2.01, *p* < 0.05) and reaction time (– 29%, *p* < 0.05) of the quadriceps muscle group better than traditional strength training (*p* < 0.05). Two studies assessed serum levels of testosterone and cortisol hormones but failed to show any effect [59, 67]. One study demonstrated a positive effect of plyometric training on capillary glucose (– 8.9% [– 8.2 mg/dL], ES 1.39, *p* < 0.001) compared with non-exercising controls [62], indicating that future researchers may wish to further investigate the effects of plyometric training in older patients with diabetes or pre-diabetes. Finally, one study assessed various kinematic, biomechanical, and muscle architectural variables, but its outcomes are unclear [66]. Therefore, the lack of studies and incongruous results of these studies do not allow for conclusive statements to be made regarding the effects of plyometric training on these isolated variables; future research should consider investigating them further.

#### Safety

Of the 289 subjects who actively participated in exercise programs that included plyometric components, only a maximum of 1.4% incurred an injury that resulted in the subject dropping out of the study. Therefore, data extracted from the studies included in this review indicate that plyometric training is likely safe to perform in older adults, especially under supervised conditions. Though only two studies included a home-based component, we may also assume that practicing plyometric training at home does not incur a significantly increased risk for the older adults, an assumption that is consistent with a previous study of high-speed training under low-supervision conditions in older women [84]. However, as stated in Sect. 3.3, the studies included in this review included mostly healthy subjects, meaning that future research should be conducted on less healthy subjects to determine whether these benefits translate across populations or if additional benefits of plyometric exercise can be identified in specific populations. Nevertheless, the data from the included studies indicate that with proper instruction, and possible supervision, the traditional strength pre-requisites that were established for athletes (i.e., the ability to perform a back squat with 1.5–2.0 times bodyweight) [40, 41] may not apply in older adults, and that basic movement competency followed by periodized progression is likely sufficient for healthy older adults.

### Strengths and Limitations

The clear strength of our review is the elaborate search strategy that, rather than relying on searching only for the term “plyometric”, combined various related terms describing potentially plyometric exercises, such as “hopping” or “jumping”. This strategy required all the papers resulting from the searches to be carefully studied to make sure that the exercises truly were plyometric. This approach proved worthwhile, as many of the papers ultimately included in this review did not contain the term “plyometric” and would not have otherwise been found. Interestingly, a recent large scoping review of plyometric jump training up to April 2017 [39] that limited the search to “plyometric” identified 242 eligible papers, but only one of them included subjects over 65 years old [64]. That being the case, future reviews of plyometric training should consider using the search strategy employed in our review instead of relying on just the “plyometric” term.

We also understand that some may view the lack of meta-analysis of the effects of plyometric training on key outcomes as a limitation of the present review. Unfortunately, the limited number of eligible studies and the heterogeneity of the outcomes and assessment methods did not allow for a meaningful meta-analysis at this time, as the number of studies available for each outcome were too low. In addition, the already small number of available studies was further fragmented by the design of the control group. For example, the isolated effect of plyometric training in comparison with the non-exercising group could be evaluated in only three studies [52, 65]. Other studies either compared plyometric training with a different form of exercise or they included plyometric exercises only as a part of multicomponent training.

## Conclusion

To summarize, the evidence supporting the effects of plyometric training on various outcomes in older adults shows that plyometric exercise may positively affect any of these outcome variables, at least when comparing the pre- to post-intervention results. Moreover, in some of the outcome categories, such as muscular strength, jump performance, and physical performance, the effects of plyometrics are more convincing and are consistently greater than those of non-exercising controls. However, only in a few cases was plyometric training superior to another type of training with similar volume and intensity, namely in performance during counter-movement jumps, the 30-s sit-to-stand test [63], and in the rate of torque development during contractions of the quadriceps femoris [67]. Importantly, plyometric training was demonstrated to be a safe training option in older adults. Therefore, plyometric training can be considered as a feasible and safe alternative to traditional strength training in older adults, especially when supervised training is designed to increase an individual’s dynamic neuromuscular performance.
